# Attitudes towards Telemedicine Services and Associated Factors among health professionals in Ethiopia: a systematic review and meta-analysis

**DOI:** 10.1186/s12913-024-11979-w

**Published:** 2024-11-29

**Authors:** Alex Ayenew Chereka, Gebrehiwot Berie Mekonnen, Amlaku Nigusie Yirsaw, Berihun Agegn Mengistie, Eyob Getachew, Gebeyehu Lakew, Adamu Ambachew Shibabaw, Gemeda Wakgari Kitil

**Affiliations:** 1 Department of Health Informatics, College of Health Sciences, Mattu University, Mattu, Ethiopia; 2 Departments of Midwifery, College of Health Sciences, Mattu University, Mattu, Ethiopia; 3 Department of Pediatrics and Child Health Nursing, College of Health Sciences, Debre Tabor University, Debre Tabor, Ethiopia; 4 Department of Health Promotion and Health Behavior, Institute of Public Health, College of Medicine and Health Sciences, University of Gondar, Gondar, Ethiopia; 5 Department of General Midwifery, School of Midwifery, College of Medicine and Health Sciences, University of Gondar, Gondar, Ethiopia

**Keywords:** Attitude, Telemedicine, Health professionals, Services, Ethiopia

## Abstract

**Background:**

Telemedicine is a vital tool for improving healthcare delivery in Ethiopia, where geographic, economic, and infrastructural challenges limit access to care, particularly in rural areas. With a shortage of healthcare professionals and limited medical services, telemedicine offers a solution by enabling remote consultations and continuous monitoring, extending healthcare to underserved populations. However, the successful adoption of telemedicine depends largely on the attitudes of healthcare professionals, whose acceptance and use of the technology are crucial for its integration. This study reviews the factors influencing Ethiopian health professionals’ attitudes toward telemedicine to provide insights that can support its adoption and improve healthcare delivery in the country.

**Methods:**

Following the PRISMA guidelines, we conducted a systematic review of studies on telemedicine attitudes, initially identifying 15,900 articles. After screening, 5 full-text articles were selected for inclusion. The data were analyzed using STATA version 11, where heterogeneity was assessed using the I² test, and publication bias was evaluated through funnel plots and Egger’s regression. The pooled effect size was calculated using a random-effects model, with a 95% confidence interval to ensure the robustness and precision of the findings.

**Results:**

The finding that 53.42% (95% CI: 42.02–64.83) of Ethiopian health professionals hold positive attitudes towards telemedicine suggests a moderate level of acceptance. Factors associated with these positive attitudes included receiving computer training in telemedicine (AOR 4.47, 95% CI: 1.94–10.26), possessing advanced digital literacy (AOR 4.08, 95% CI: 1.30–12.81), comprehensive knowledge of telemedicine technology (AOR 3.28, 95% CI: 1.73–6.23), access to reliable internet (AOR 3.04, 95% CI: 1.67–5.53), and availability of electronic devices in healthcare settings (AOR 2.59, 95% CI: 1.73–3.87).

**Conclusion and recommendations:**

This meta-analysis reveals that 53.42% of Ethiopian health professionals hold positive attitudes towards telemedicine, influenced by specialized training, digital literacy, and resource access. To enhance adoption, initiatives should focus on targeted training, reliable internet connectivity, availability of devices in healthcare settings, and promoting broader awareness about telemedicine benefits and applications among healthcare professionals.

**Supplementary Information:**

The online version contains supplementary material available at 10.1186/s12913-024-11979-w.

## Introduction

Technological advancements have played a crucial role in transforming many industries, including healthcare [1]. The incorporation of cutting-edge technologies in the medical sector has greatly advanced medical progress and changed the way healthcare services are provided [2–5]. The modern healthcare system uses advanced information technologies to improve care delivery and promote health. Tools like health information websites, online support networks, electronic medical records, and mobile health devices can help close the healthcare gap between urban and rural areas [6]. These technologies are essential for capturing, storing, processing, analyzing, and communicating healthcare information. Understanding the factors that influence individuals’ acceptance of healthcare technology is crucial. Telemedicine, in particular, represents a significant advancement in healthcare, enabling enhanced collaboration among medical practitioners, hospitals, health centers, and insurance experts within a digital framework. This collaborative approach aims to improve the equity of medical service distribution, enhance service quality, and reduce costs, especially during public health crises [7–9].

The rapid rise and influence of telemedicine, which involves delivering healthcare services remotely via telecommunication networks, has marked it as a pivotal innovation in the healthcare sector [10]. By enhancing medical care accessibility, improving patient convenience, and potentially lowering costs, telemedicine has become increasingly significant [11].Telemedicine refers to the use of digital communication technologies, such as video conferencing, remote monitoring devices, and telehealth platforms, to provide healthcare services and exchange medical information over distances [12].

This includes real-time consultations between patients and healthcare professionals, remote diagnostic services, virtual follow-ups, and continuous health monitoring using digital devices that track vital signs or manage chronic conditions. Telemedicine also encompasses the use of electronic health records (EHR) for sharing patient information securely, as well as the delivery of health education and prevention programs through online platforms. By facilitating communication and access to healthcare, telemedicine can bridge the gap for individuals in remote or underserved areas who may otherwise lack access to specialized medical services [13, 14]. Telemedicine is seen as a promising solution for medical challenges in less developed countries by enabling the transfer of expert medical knowledge to remote areas where it’s needed.

Telemedicine in Ethiopia has seen several initiatives aimed at overcoming access barriers and strengthening healthcare delivery, particularly in rural areas. The Ethiopian Ministry of Health’s “Digital Health Strategy 2020–2025” prioritizes telemedicine to improve access to care and develop digital infrastructure in underserved areas [15]. The Ethiopian Telemedicine Project, developed in collaboration with the World Health Organization (WHO), connects rural health centers with urban hospitals, enabling specialist consultations for remote populations who otherwise face limited access to advanced medical care [16, 17].

These initiatives have also extended to providing remote medical education for healthcare workers in underserved regions, helping bridge the skills gap in areas lacking specialized training. However, the success of telemedicine integration largely depends on healthcare providers’ perceptions and acceptance of telemedicine as a viable healthcare model. Studies indicate that factors such as internet access, the availability of supportive infrastructure, and provider training play crucial roles in shaping these attitudes [18]. Efforts to address these factors are essential to ensure the effective adoption of telemedicine across Ethiopia and to improve healthcare access for isolated communities.

However, its adoption in Ethiopia and other developing nations has been limited, with minimal usage reported [19, 20]. Additionally, telemedicine promotes preventive healthcare, benefiting individuals who face barriers to quality care due to financial or geographic constraints [21, 22]. The successful integration of telemedicine into healthcare systems hinges significantly on how healthcare providers perceive and approach it. Therefore, gaining a deep understanding of these attitudes is essential for the effective implementation of telemedicine technologies [23, 24].

Research on healthcare providers’ attitudes towards telemedicine is significantly influenced by their access to technology and reliable internet connectivity. The availability of necessary infrastructure is crucial for facilitating telemedicine services and shaping providers’ acceptance of this healthcare delivery method [25]. Improved connectivity also promotes collaborations among healthcare professionals, enabling them to form support networks that enhance overall healthcare provision. Despite initial technological challenges and skepticism, telemedicine remains a potent tool that complements and improves the patient experience [13, 23, 26].

Research on healthcare providers’ attitudes towards telemedicine shows a nuanced interaction between personal traits like age, gender, and professional experience, and organizational factors such as technological infrastructure, training, support systems, and reimbursement policies [27]. Empirical data indicates that providers’ acceptance of telemedicine is influenced by their expectations of effort, attitudes toward technology, self-confidence in using it, expectations of performance, and the presence of supportive conditions [2, 28]. Research shows that healthcare professionals’ acceptance of telemedicine is influenced by factors like their expectations of effort, attitude toward technology, confidence in using it, and expected performance benefits. This systematic review and meta-analysis seeks to comprehensively evaluate healthcare providers’ attitudes toward telemedicine and explore the factors influencing these attitudes. By synthesizing data from relevant studies, the analysis will provide critical insights into the current state of telemedicine acceptance among healthcare professionals, highlighting both the enabling factors and barriers. These insights will inform the development of targeted strategies to enhance telemedicine implementation, facilitating its integration into healthcare systems and improving access to care. Moreover, the findings can guide policymakers, health planners, and both governmental and non-governmental organizations in shaping policies and interventions that promote telemedicine adoption, ultimately contributing to the improvement of healthcare delivery and access in Ethiopia.

## Methods and materials

### Source of information and search strategy

This systematic review and meta-analysis, titled “Health Professionals’ Attitudes towards Telemedicine Services and Associated Factors in Ethiopia,” focuses on health professionals in Ethiopia as the population of interest (P). The study examines the attitudes towards telemedicine services among these professionals (I). Since this is a systematic review and meta-analysis, no specific comparison group is identified; instead, the primary objective is to synthesize findings across studies. The main outcome of interest is to identify and analyze factors associated with health professionals’ attitudes towards telemedicine services in Ethiopia (O).

To find relevant research, we thoroughly searched the PROSPERO database and the Database of Abstracts of Reviews of Effects (DARE) on the UCSF library website. Our study, adhering to the PRISMA guidelines [29], (S1 Table).We conducted a systematic search across a comprehensive range of academic databases, including Google Scholar, PubMed, HINARI, Web of Science, Global Health, Scopus, and the African Journal Online (AJOL), from May 5 to July 5, 2024. This search aimed to identify all relevant literature related to our research topic, with no restrictions on publication date. By allowing for the inclusion of studies from any publication year, we ensured a broad scope in capturing pertinent findings, regardless of when the research was originally published. This approach maximizes the likelihood of obtaining a thorough and representative sample of available literature on the attitudes of health professionals toward telemedicine and related factors.

Our search utilized a combination of keywords, free-text search queries, and Medical Subject Headings (MeSH). Boolean operators were employed to combine terms related to (“Attitude” AND “Telemedicine” OR “telehealth” OR “tele psychiatry” OR “tele education” OR “tele surgery” OR “tele radiology” AND “health professional” OR “healthcare provider” OR “healthcare workers” OR “Physicians” OR “Nurses” AND “associated factors” OR “determinants “OR “influences factors” AND “Ethiopia”). This methodical approach ensured a comprehensive and structured review of the literature during the specified timeframe.

### Eligibility criteria

This systematic review investigated healthcare professionals’ attitudes towards telemedicine services in Ethiopia, examining associated factors through original observational research, including cohort, cross-sectional, and case-control studies. To ensure a broad and current analysis, we placed no restrictions on the publication year of the studies. Only full-text, peer-reviewed articles in English were selected to maintain consistency, accessibility, and adherence to high scientific standards. English-language studies were chosen due to their established methodological rigor and global accessibility, facilitating replicability and wider dissemination. To prevent bias and ensure consistent data extraction, we excluded studies in languages other than English.

Furthermore, we omitted non-cross-sectional studies, such as case reports, conference proceedings, expert opinions, editorials, reviews, letters, and commentaries, as these typically lack the methodological rigor and generalizability necessary for meta-analysis.

Our study included a broad sample of healthcare professionals, comprising 877 nurses, 642 physicians, 153 psychiatrists, 38 medical laboratory technicians, 32 midwives, 29 pharmacists, 15 health officers, and various other practitioners. This diverse representation allows for a comprehensive understanding of telemedicine attitudes across different healthcare roles, offering valuable insights for supporting telemedicine integration in Ethiopia.

### Appraisal and quality assessment

This study employed a rigorous appraisal and quality assessment of the included studies to ensure the reliability and validity of the findings. Each study was evaluated using established criteria to assess methodological quality, risk of bias, and relevance to the research question. We utilized the Joanna Briggs Institute Critical Appraisal Checklists to systematically review aspects such as study design, adequacy of sample size, data collection methods, and consistency of results. This comprehensive assessment ensures the inclusion of high-quality studies, enhancing the robustness of our systematic review and meta-analysis findings (S2 Table)*.*

Given the inclusion of fewer than 10 studies, we recognize that publication bias tests, such as funnel plots and Egger’s regression, may not yield definitive results due to the small sample size. The limited number of studies may reduce the sensitivity and reliability of these tests, meaning the results should be interpreted with caution. Despite this limitation, the pooled prevalence and the associated factors provide valuable insights into the attitudes toward telemedicine among healthcare professionals in Ethiopia.

### Data extraction process

We utilized the Joanna Briggs Institute (JBI) cross-sectional study quality assessment checklist to evaluate the studies’ quality [30]. Data extraction was conducted by two authors (GWK and AAS) using a pre-established checklist in Microsoft Excel blindly. This process aimed to gather essential information, including author names, publication year, study location, design, sample size, prevalence estimates, response rate, and involvement of Health professionals.

We began by consolidating search results from multiple databases to streamline the process. Using EndNote version 20.0, we then identified and removed duplicate articles. Next, we carefully reviewed study titles and abstracts to exclude irrelevant information. Full-text publications underwent a thorough assessment to select the most relevant research articles. Reviewers AAC and GWK resolved disagreements through collaborative discussions to reach a consensus. When primary publications lacked sufficient information, we contacted corresponding authors via email for clarification.

### Evaluation of attitudes towards telemedicine

In this systematic review and meta-analysis, attitudes toward telemedicine were evaluated using various measurement tools across studies, including Likert scales, percentage-based responses, and binary categories. Positive attitudes were defined as mean scores above the midpoint of a scale (e.g., > 3 on a 5-point scale) or when more than 50% of respondents expressed favorable views. In studies with binary responses (e.g., agree/disagree), attitudes were classified as positive if the majority of respondents agreed with telemedicine-related statements. The aspects of attitudes considered included perceived usefulness, ease of use, openness to technology integration, and perceived barriers such as resource limitations and training deficiencies. Variations in measurement tools across studies were standardized, ensuring consistency in the interpretation of results for meta-analysis.2.6 Outcome measurement.

The primary outcome of this study is to assess the attitudes of Ethiopian health professionals toward telemedicine services. This assessment includes measuring their acceptance, willingness to use telemedicine, and perceptions of its benefits and challenges. To quantify these attitudes, structured questionnaires and standardized scales, such as Likert scales, are employed, enabling a detailed evaluation of factors like perceived usefulness, ease of use, and overall readiness to adopt telemedicine.

In addition to attitudes, the study explores various factors that influence these perceptions, including demographic characteristics and work-related conditions. Many studies indicate perceived benefits of telemedicine, such as enhanced quality of care and improved accessibility for patients, while also noting challenges, including limited resources, technological barriers, and a lack of training. This comprehensive approach aims to provide insights into both the positive and negative aspects of telemedicine as viewed by health professionals in Ethiopia.

### Synthesis and analysis of data

Data extraction and synthesis were performed using Microsoft Excel. The retrieved data were analyzed using STATA version 14. Tables, forest plots, and figures were utilized to comprehensively describe and summarize the primary studies. To assess the overall attitude of health professionals toward telemedicine services, a random-effects model with a 95% confidence interval (CI) was employed.

We assessed the impact of variables on Health Professionals’ attitudes towards Telemedicine Services using odds ratios with 95% confidence intervals. Given the substantial variability among the included studies, a random-effects model was employed in the meta-analysis to ensure thorough examination and interpretation of all data.

To quantify the variation in reported attitudes across studies, Cochran’s Q test and I2 statistics were utilized. Statistical significance for Cochran’s Q test was determined by a *p*-value less than 0.05. The I2 statistic ranges from 0 to 100%, indicating no (0%), modest (25%), moderate (50%), or large (75%) degree of variance. Furthermore, Egger regression tests and funnel plot shape analyses were conducted to assess potential publication bias.

## Results

### The process of selecting articles

**Fig. 1:**
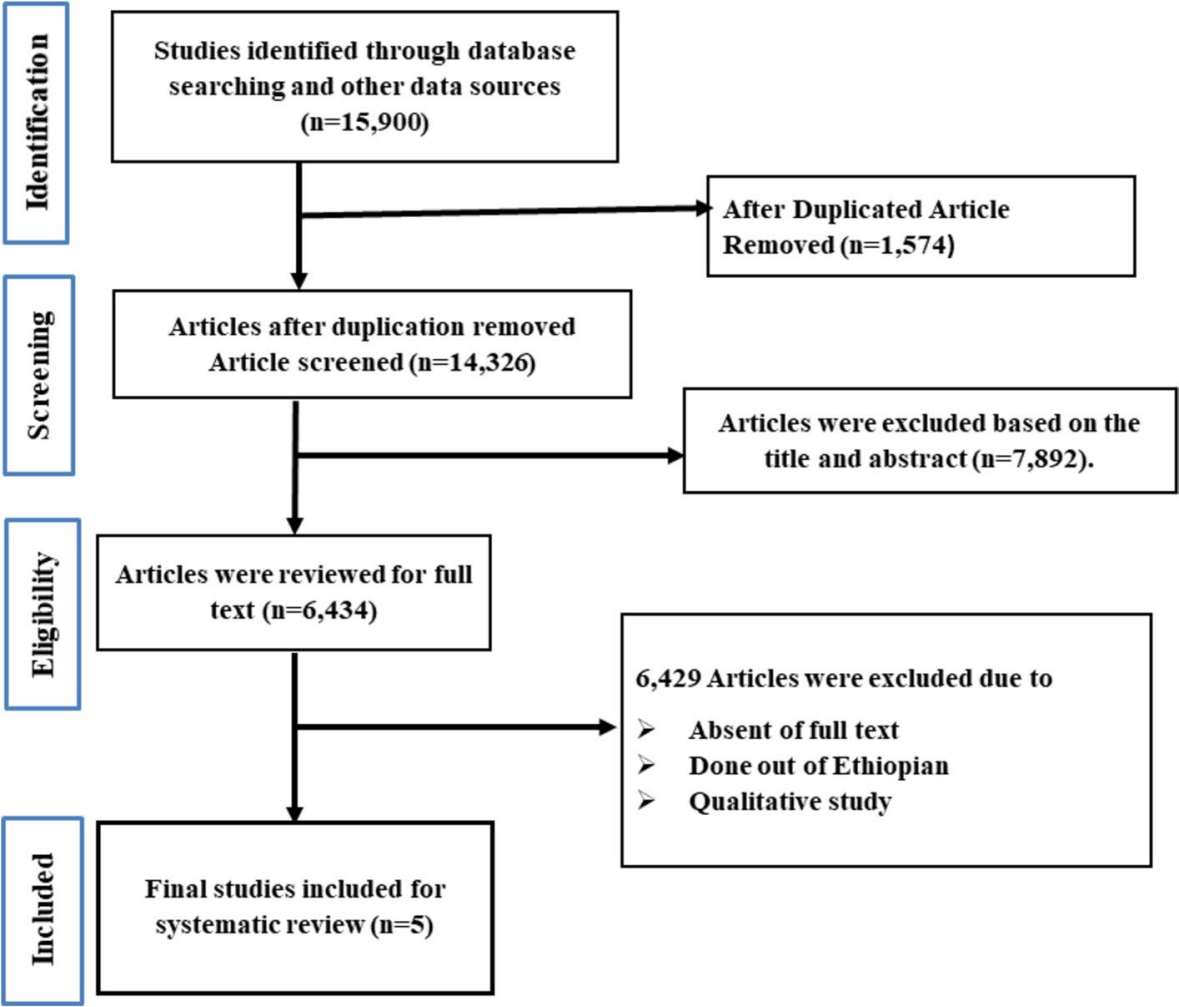
The PRISMA flowcharts illustrate the process of selecting articles for inclusion in this study

We conducted a comprehensive search across various databases and search engines, initially identifying 15,900 articles. After removing 1,574 duplicates, we were left with 14,326 unique articles. These articles were screened based on their titles and abstracts, leading to the exclusion of 7,892 that did not match our study focus. Subsequently, we performed a detailed full-text review of the remaining articles, excluding an additional 5,945 that were unrelated to our study area. Additionally, 484 articles were excluded due to unavailable full texts. Ultimately, after this rigorous screening process, we identified 5 full-text articles that met our inclusion criteria for the systematic review and meta-analysis. This meticulous method ensured that only the most relevant studies were included in our analysis (Fig. 1).

### The included articles’ characteristics in this review

This research, involving five articles and 2,031 participants, evaluated health professionals’ attitudes toward telemedicine and associated factors in Ethiopian healthcare institutions. The study’s significance lies in its potential to inform policy and training programs to enhance telemedicine adoption, thereby improving healthcare delivery in Ethiopia. Each article provided valuable insights into the current state of telemedicine attitudes and associated factors.

In this meta-analysis, over three-fourths of the studies were conducted in the Amhara region, involving 1,581 participants [31–34]. This concentration highlights regional disparities in telemedicine attitudes and the need for targeted interventions. The studies in Amhara offered a comprehensive overview of the challenges and opportunities related to telemedicine, reflecting the region’s unique healthcare dynamics and infrastructural limitations.

The remaining studies were conducted in Addis Ababa, involving 450 participants [35]. As the capital and largest urban center, Addis Ababa presents different circumstances compared to the Amhara region. Studies from Addis Ababa focused on the urban healthcare setting, where technological infrastructure and access to resources are relatively better. These studies highlighted the potential for telemedicine in urban environments and the challenges faced by health professionals, including training, technology adoption, and institutional support (Table 1).

**Table 1:** Individual characteristics of selected articles

Authors	Region	study year	publication year	Study design	Study Populations	sample size	Prevalence	Quality score
Sidelil H et al.	Amhara	2021	2023	cross-sectional	Health professionals	423	69.9	9
Seboka B. T et al.	Amhara	2018	2018	cross-sectional	Health professionals	312	64	8
Butta F. W et al.	Amhara	2023	2024	cross-sectional	Nursing	423	39.7	9
Adem J. B et al.	Addis Ababa	2022	2023	cross-sectional	Healthcare providers	450	51	8
Reda M. M et al.	Amhara	2022	2024	cross-sectional	Physicians	423	43.1	8

### The pooled prevalence of health professionals’ attitude towards telemedicine technology in Ethiopia

The meta-analysis found that 53.42% (95% CI: 42.02–64.83) of Ethiopian health professionals demonstrate a positive attitude toward telemedicine technology, representing an important foundation for telemedicine integration within Ethiopia’s healthcare system. This level of acceptance highlights a substantial portion of healthcare providers open to adopting telemedicine, which can be leveraged in developing targeted initiatives to foster greater utilization and support. This positive baseline is essential for building engagement strategies that address specific barriers to adoption across different professional groups and settings.

**Fig. 2:**
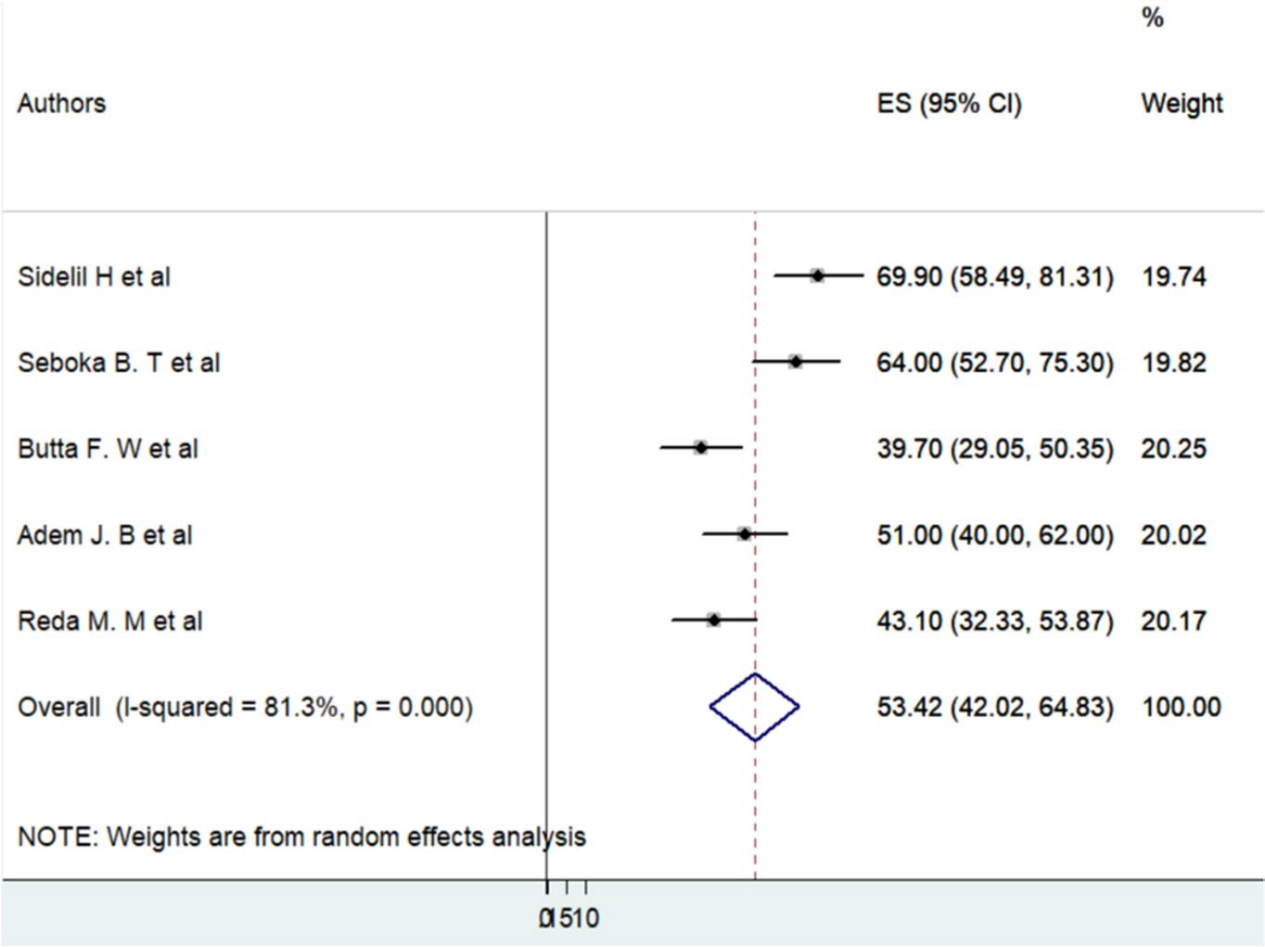
A forest plot of the pooled prevalence of Health Professionals’ attitude towards telemedicine technology in Ethiopia

However, the analysis also identified significant heterogeneity (I² = 81.3%; *p* = 0.00), indicating that there is considerable variation in attitudes across the studies included. This level of heterogeneity suggests that factors such as variations in study populations, research methods, or healthcare settings may impact professionals’ attitudes towards telemedicine. To address this variability, conducting subgroup analyses will be important to uncover the factors driving differences in attitudes, providing insights for customized interventions that can optimize telemedicine adoption across Ethiopia’s healthcare workforce (Fig. 2).

### Publication bias

**Fig. 3:**
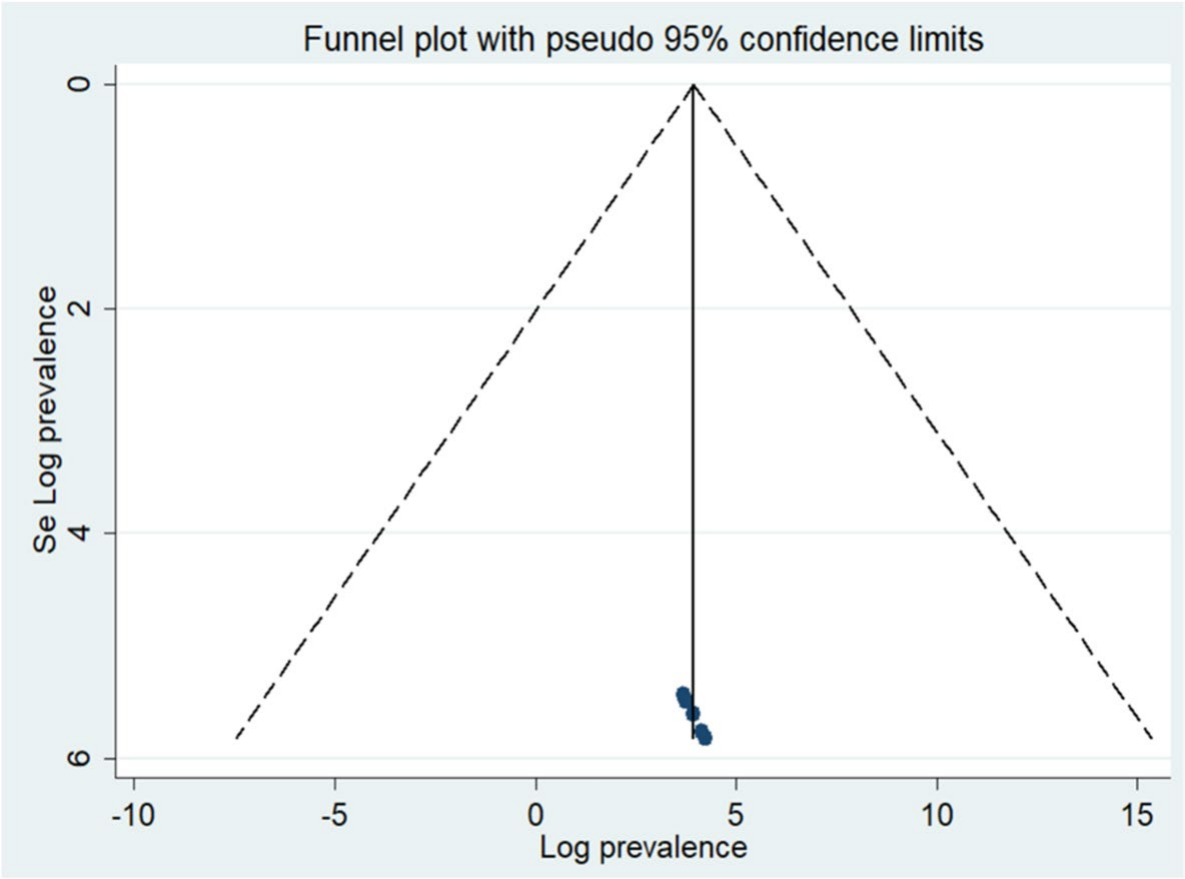
A funnel plot of the included studies, assessing publication bias

To assess publication bias in our meta-analysis, we employed two techniques: Egger’s regression test and a visual examination of a funnel plot. Initially, the funnel plot, which plots study precision against effect size, showed an asymmetric distribution, raising some concerns. Nevertheless, Egger’s regression test resulted in a non-significant finding (*P* = 0.21), indicating no significant evidence of publication bias. This statistical outcome, corroborated by the visual evaluation of the funnel plot, reinforces the reliability of our meta-analysis results. It suggests that the included studies were not systematically biased, as depicted in Fig. 3.

### Subgroup analysis

**Fig. 4:**
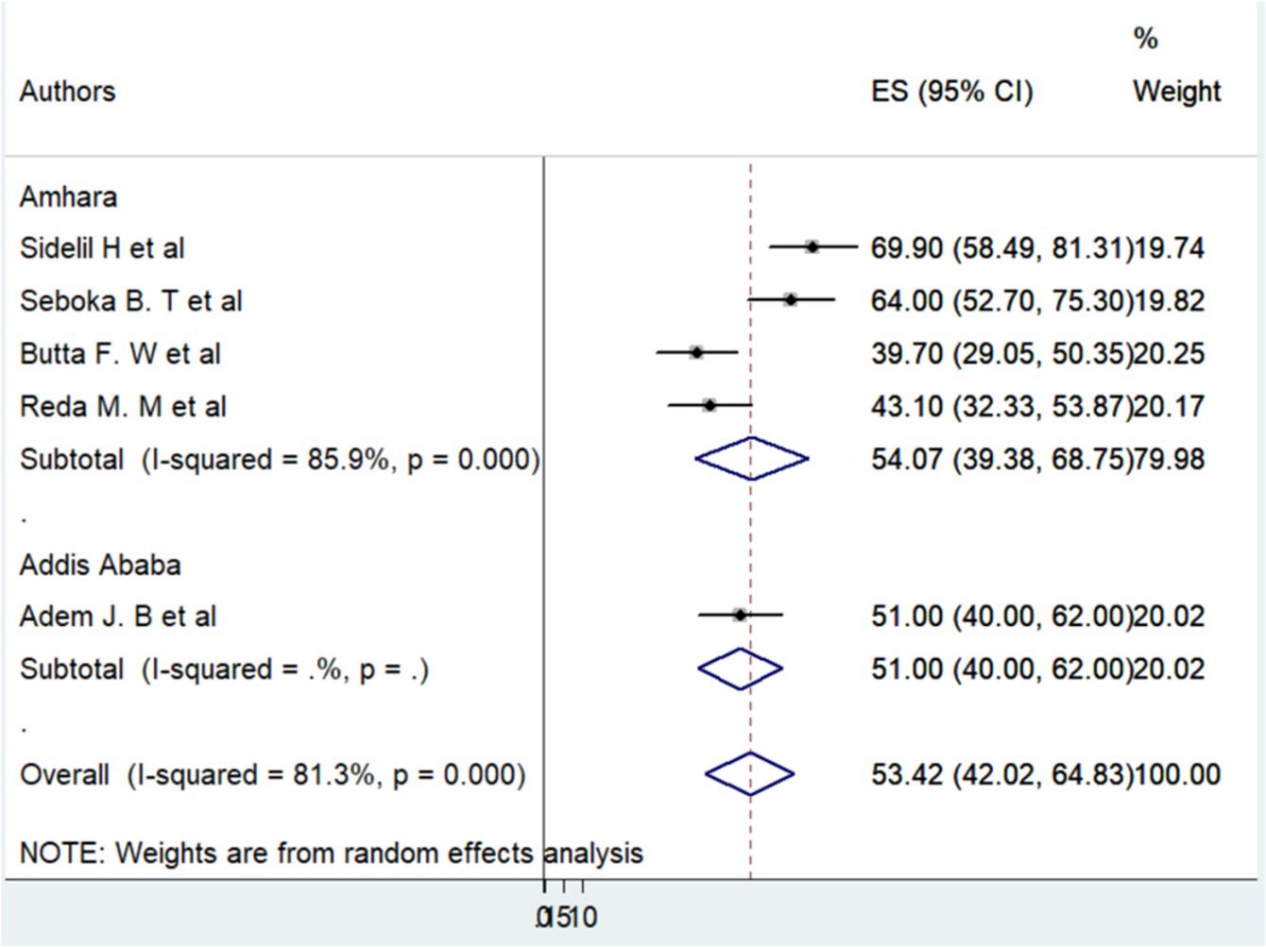
Subgroup meta-analysis of factors related to heterogeneity in health professionals’ awareness of telemedicine in Ethiopia

In this meta-analysis, there was noticeable diversity among the studies reviewed. To explore this variation, researchers conducted a subgroup analysis considering factors like where the studies were conducted, how many people were involved, when they were published, how many people responded, and the timeframe of the studies. The analysis showed that studies from the Amhara region and those with smaller participant groups were major reasons for the differences seen across studies (I2 = 85.9%, *p* = 0.00). Additionally, studies with lower response rates also contributed significantly to these differences (I2 = 55.3%, *p* = 0.056). However, when looking at when studies were published and their study periods, these factors didn’t seem to impact the differences observed. These findings highlight the complex and evolving nature of attitudes towards telemedicine, influenced by diverse local situations and changes in healthcare practices (Fig. 4).

### Factors associated with health professionals’ attitude of telemedicine in Ethiopia

Health professionals’ attitudes toward telemedicine in Ethiopia are influenced by several important factors. Research shows that those who have received computer training are more likely to adopt telemedicine technologies (AOR 4.47, 95% CI: 1.94–10.26). Similarly, health professionals with strong digital literacy are more likely to have a positive attitude toward telemedicine (AOR 4.08, 95% CI: 1.30–12.81), emphasizing the role of digital skills in supporting adoption.

**Fig. 5:**
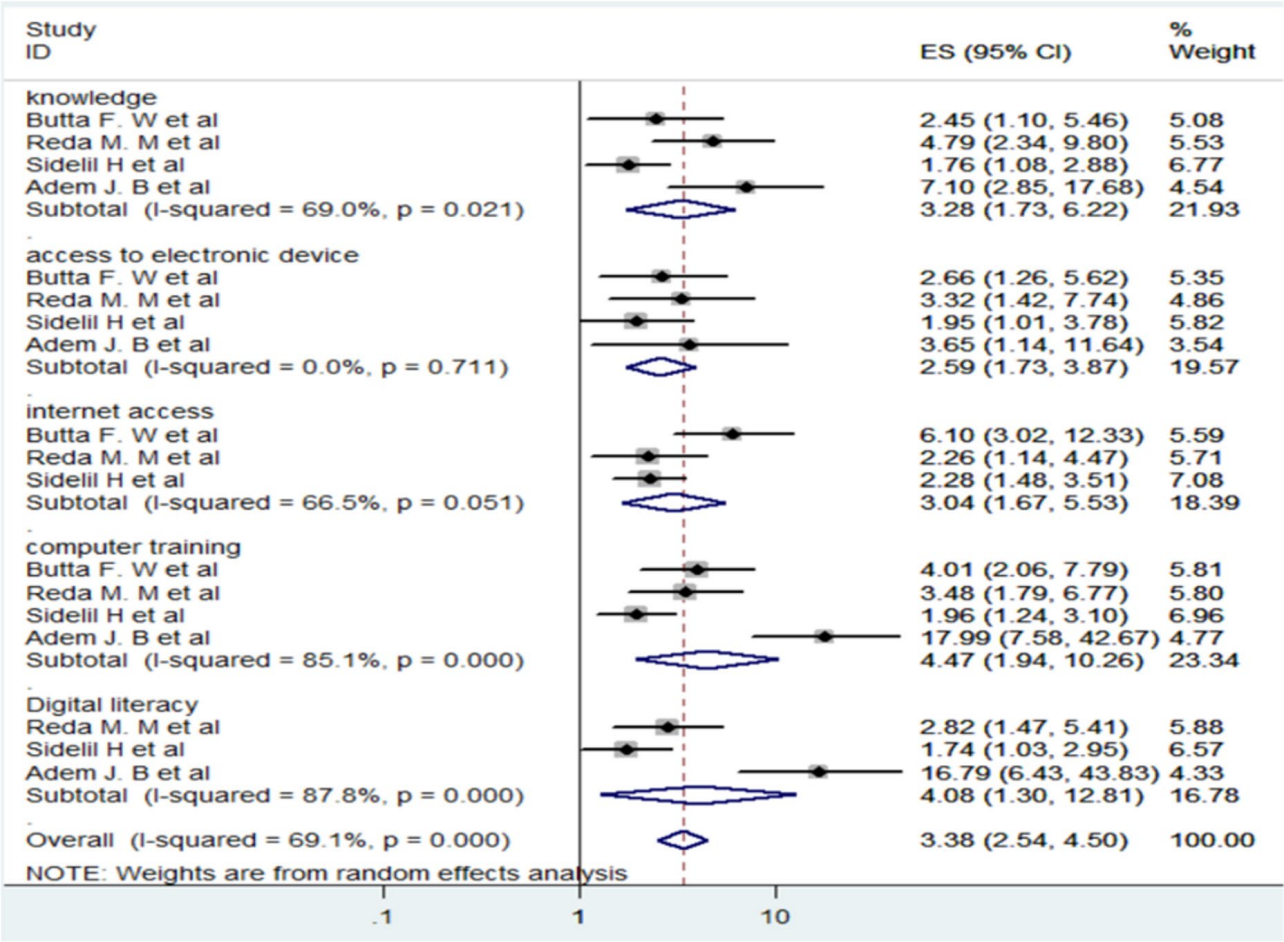
Forest plot of factors associated with health professionals’ awareness of telemedicine in Ethiopia

In addition, having a good understanding of telemedicine technology is associated with more favorable attitudes (AOR 3.28, 95% CI: 1.73–6.23). Reliable internet access (AOR 3.04, 95% CI: 1.67–5.53) and the availability of electronic devices in healthcare settings (AOR 2.59, 95% CI: 1.73–3.87) also play a key role in shaping positive attitudes. These factors help integrate telemedicine more effectively into clinical practice, making it easier for health professionals to adopt and use telemedicine technologies (Fig. 5).

## Discussion

Telemedicine has emerged as a powerful means of enhancing healthcare delivery, particularly in resource-limited settings such as Ethiopia [36]. The country faces significant challenges in delivering quality healthcare, including limited access to services, geographic barriers, and shortages of healthcare professionals, which often lead to inequities in healthcare access, particularly in rural and underserved regions [37]. In this context, telemedicine offers a promising solution by facilitating remote consultations, diagnostics, and continuous patient monitoring, thereby addressing logistical and infrastructural barriers that impede effective healthcare delivery [38, 39]. Telemedicine can significantly reduce the need for patients to travel long distances for medical care and offer health professionals a way to extend their services beyond traditional face-to-face encounters [40]. The successful implementation and widespread adoption of telemedicine in Ethiopia, and in similar low-resource settings, depend not only on the technology itself but also on the attitudes of healthcare professionals, who play a crucial role in its integration into the healthcare system [41]. Health professionals’ attitudes toward telemedicine are shaped by several key factors, including computer training, digital literacy, comprehensive understanding of telemedicine, access to electronic devices in healthcare settings, and reliable internet connectivity. Studies indicate that computer training, in particular, significantly enhances health professionals’ willingness to adopt and integrate telemedicine technologies into clinical practice [23].

This systematic review and meta-analysis aimed to assess the attitudes of Ethiopian health professionals toward telemedicine, with a focus on identifying factors that influence these perspectives. The findings indicated that 53.42% (95% CI: 42.02–64.83) of Ethiopian health professionals reported a positive attitude toward telemedicine, reflecting a moderate level of acceptance within the healthcare workforce [31]. While this level of acceptance is promising for the future of telemedicine adoption, the moderate proportion of positive attitudes also reveals considerable variability among different health professionals. This variability underscores the importance of targeted efforts to understand the factors driving differing attitudes and to address the barriers to broader acceptance. The results highlight the need to bridge gaps in knowledge, training, and access to technology in order to strengthen telemedicine integration into the healthcare system in Ethiopia [39, 42].

Based on the analysis of associated factors, five factors were significantly linked to attitudes towards telemedicine technology. Among these, health professionals in Ethiopia who received computer training were significantly more likely to exhibit positive attitudes towards telemedicine, with an Adjusted Odds Ratio (AOR) of 4.47. This finding is supported by a study conducted in Saudi Arabia [43]. It suggests that training programs enhance technical proficiency, equipping professionals with the skills needed to effectively navigate and utilize telemedicine platforms. Such proficiency reduces uncertainties and boosts confidence in integrating telemedicine into clinical practice.

Secondly, advanced digital literacy among respondents was strongly associated with favorable attitudes towards telemedicine technologies, with an AOR of 4.08. Digital literacy empowers healthcare professionals to adeptly use digital tools, increasing their comfort and efficiency in telemedicine applications. This familiarity fosters trust in the reliability and effectiveness of telemedicine solutions, thereby promoting acceptance and adoption.

Moreover, comprehensive knowledge of telemedicine technology significantly influenced attitudes, with an AOR of 3.28. Healthcare professionals who possess a deep understanding of telemedicine functionalities and benefits perceive it as a valuable tool for enhancing patient care and expanding access to healthcare services. This knowledge promotes a proactive approach towards integrating telemedicine into healthcare delivery, driven by the clear benefits it offers in overcoming geographical barriers and improving healthcare outcomes.

Additionally, access to reliable internet connectivity emerged as a critical factor influencing attitudes towards telemedicine, with an AOR of 3.04. Reliable internet access facilitates seamless communication, data exchange, and teleconsultations, which are essential for effective telemedicine practice. Professionals with dependable internet connectivity can confidently engage in remote consultations and collaborate across distances, thereby recognizing the practical advantages of telemedicine in expanding healthcare access.

Lastly, the availability of electronic devices within healthcare organizations significantly contributed to positive attitudes towards telemedicine, with an AOR of 2.59. Access to devices such as computers and smartphones enables healthcare professionals to access telemedicine platforms easily and provide remote care efficiently. This finding is supported by a study conducted in Saudi Arabia [43]. This accessibility eliminates logistical barriers and supports the seamless integration of telemedicine into daily practice, reinforcing positive perceptions and acceptance among healthcare providers.

### Strengths and limitations

This systematic review and meta-analysis provides a comprehensive and robust examination of health professionals’ attitudes towards telemedicine services in Ethiopia, identifying key factors that influence these attitudes. The synthesis of data from multiple studies enhances the reliability of the findings, offering valuable insights for local policy and decision-making. However, the study’s findings may have limited generalizability outside Ethiopia, and potential publication bias could affect the results. Variability in the quality and design of included studies, along with data heterogeneity, might complicate interpretation. Furthermore, the review might not fully capture recent developments and changes in telemedicine adoption due to its reliance on previously published studies.

## Conclusion

This systematic review and meta-analysis highlight the multifaceted determinants influencing health professionals’ attitudes toward telemedicine technologies in Ethiopia. Addressing factors such as education, digital literacy, knowledge enhancement, internet accessibility, and technological infrastructure is crucial for promoting widespread acceptance and effective implementation of telemedicine initiatives in Ethiopian healthcare settings. Future efforts should focus on targeted interventions to enhance these factors and thereby improve the uptake and impact of telemedicine in the country’s healthcare landscape.

## Supplementary Information

Supplementary Material 1. PRISMA 2020 checklist (DOC).

Supplementary Material 2. Quality assessment of Attitudes towards Telemedicine Services and Associated Factors among Health Professionals in Ethiopia (DOC).

Supplementary Material 3. Data set (Factors).

Supplementary Material 4. Data set (prevalence).

## Data Availability

Availability of data All the data utilized and analyzed in this study are publicly accessible and provided in the supporting materials.
