# Supplementary material for: Attitudes towards Telemedicine Services and Associated Factors among health professionals in Ethiopia: a systematic review and meta-analysis
Source: BMC Health Serv Res. 2024 Nov 29;24:1505. doi: 10.1186/s12913-024-11979-w (PMC11606080; doi:10.1186/s12913-024-11979-w)
Supplement: Supplementary file 2 — Supplementary Material 2. Quality assessment of Attitudes towards Telemedicine Services and Associated Factors among Health Professionals in Ethiopia (DOC). [file 12913_2024_11979_MOESM2_ESM.docx]

**S2 Table: Quality assessment of Attitudes towards Telemedicine Services and Associated Factors among Health Professionals in Ethiopia.**

| **Authors** | **Theoretical approach** | | **Data collection** | **Study design** | **Validity** | | **Analysis** | | | | **Ethics** | | **Overall quality** | **First reviewer** | **Checker** |
| --- | --- | --- | --- | --- | --- | --- | --- | --- | --- | --- | --- | --- | --- | --- | --- |
|  | Is a qualitative approach appropriate? | Is the study clear in what it seeks to do? | How well was the data collection carried out? | How defensible/rigorous is the research design/methodology | Is the context clearly described? | Were the methods reliable? | Are the data ‘rich’? | Is the analysis reliable? | Are the findings convincing? | Are the conclusions adequate? | Was the study approved by an ethics committee? | Is the role of the researcher clearly described? |  |  |  |
| Sidelil H et al | Appropriate | Clear | Adequately | Defensible | Clear | Reliable | Rich | Reliable | Convincing | Adequate | Yes | not reported | Medium | GWK | AAC |
| Seboka B. T et al | Appropriate | Clear | Adequately | Defensible | Clear | Reliable | Rich | Reliable | Convincing | Adequate | Yes | Not clear | Medium | GWK | AAS |
| Butta F. W et al | Appropriate | Unclear | adequately | Not defensible | Clear | Uncertain | Poor | Uncertain | Not convincing | Inadequate | Not reported | Not clear | low | AAC | GWK |
| Adem J. B et al | Appropriate | Clear | Adequately | Defensible | Clear | Reliable | Uncertain | Reliable | Convincing | Adequate | Yes | Uncertain | Medium | AAS | AAC |
| Reda M. M et al | Appropriate | Clear | Adequately | Defensible | Clear | Reliable | Uncertain/not reported | Reliable | Convincing | Adequate | Yes | Yes | Medium | AAS | AAC |

**Question codes:**

1. Was the sample frame appropriate to address the target population?

2. Were study participants sampled in an appropriate way?

3. Was the sample size adequate?

4. Were the study subjects and the setting described in detail?

5. Was the data analysis conducted with sufficient coverage of the identified sample?

6. Were valid methods used for the identification of the condition?

7. Was the condition measured in a standard, reliable way for all participants?

8. Was there appropriate statistical analysis?

9. was the response rate adequate, and if not, was the low response rate managed appropriately?
